# 3' tag digital gene expression profiling of human brain and universal reference RNA using Illumina Genome Analyzer

**DOI:** 10.1186/1471-2164-10-531

**Published:** 2009-11-16

**Authors:** Yan W Asmann, Eric W Klee, E Aubrey Thompson, Edith A Perez, Sumit Middha, Ann L Oberg, Terry M Therneau, David I Smith, Gregory A Poland, Eric D Wieben, Jean-Pierre A Kocher

**Affiliations:** 1 Division of Biomedical Statistics and Informatics, Department of Health Sciences Research, Mayo Clinic, Rochester, MN 55905, USA; 2 Department of Cancer Biology, Mayo Clinic Comprehensive Cancer Center, Mayo Clinic, Jacksonville, FL 32224, USA; 3 Division of Experimental Pathology, Department of Laboratory Medicine and Pathology, Mayo Clinic, Rochester, MN 55905, USA; 4 Mayo Vaccine Research Group, the Program in Translational Immunovirology and Biodefense, Department of Medicine, Mayo Clinic, Rochester, MN 55905, USA; 5 Advanced Genomics Technology Center DNA sequencing lab, Mayo Clinic College of Medicine, Mayo Clinic, Rochester, MN 55905, USA

## Abstract

**Background:**

Massive parallel sequencing has the potential to replace microarrays as the method for transcriptome profiling. Currently there are two protocols: full-length RNA sequencing (RNA-SEQ) and 3'-tag digital gene expression (DGE). In this preliminary effort, we evaluated the 3' DGE approach using two reference RNA samples from the MicroArray Quality Control Consortium (MAQC).

**Results:**

Using Brain RNA sample from multiple runs, we demonstrated that the transcript profiles from 3' DGE were highly reproducible between technical and biological replicates from libraries constructed by the same lab and even by different labs, and between two generations of Illumina's Genome Analyzers. Approximately 65% of all sequence reads mapped to mitochondrial genes, ribosomal RNAs, and canonical transcripts. The expression profiles of brain RNA and universal human reference RNA were compared which demonstrated that DGE was also highly quantitative with excellent correlation of differential expression with quantitative real-time PCR. Furthermore, one lane of 3' DGE sequencing, using the current sequencing chemistry and image processing software, had wider dynamic range for transcriptome profiling and was able to detect lower expressed genes which are normally below the detection threshold of microarrays.

**Conclusion:**

3' tag DGE profiling with massive parallel sequencing achieved high sensitivity and reproducibility for transcriptome profiling. Although it lacks the ability of detecting alternative splicing events compared to RNA-SEQ, it is much more affordable and clearly out-performed microarrays (Affymetrix) in detecting lower abundant transcripts.

## Background

The transcriptome can be profiled by high throughput techniques including SAGE [1], microarray [2,3], and sequencing of clones from cDNA libraries [4,5]. For more than a decade, oligo-nucleotide microarrays have been the method of choice providing high throughput and affordable costs. However, microarray technology suffers from well-known limitations including insufficient sensitivity for quantifying lower abundant transcripts, narrow dynamic range and non-specific hybridizations. Additionally, microarrays are limited to only measuring known/annotated transcripts and often suffer from inaccurate annotations [6]. Sequencing-based methods such as SAGE rely upon cloning and sequencing cDNA fragments. This approach allows quantification of mRNA abundance by counting the number of times cDNA fragments from a corresponding transcript are represented in a given sample, assuming that cDNA fragments sequenced contain sufficient information to identify a transcript. Sequencing-based approaches have a number of significant technical advantages over hybridization-based microarray methods. The output from sequence-based protocols is digital, rather than analog, obviating the need for complex algorithms for data normalization and summarization while allowing for more precise quantification and greater ease of comparison between results obtained from different samples. Consequently the dynamic range is essentially infinite, if one accumulates enough sequence tags. Sequence based approaches do not require prior knowledge of the transcriptome and are therefore useful for discovery and annotation of novel transcripts as well as for analysis of poorly annotated genomes. However, until recently the application of sequencing technology in transcriptome profiling has been limited by high cost, by the need to amplify DNA through bacterial cloning, and by the traditional Sanger approach of sequencing by chain termination [7].

The next-generation sequencing (NGS) technology [8-10] eliminates some of these barriers, enabling massive parallel sequencing at a high but reasonable cost for small studies. The technology essentially reduces the transcriptome to a series of randomly fragmented segments of a few hundred nucleotides in length. These are amplified by a process that retains spatial clustering of the PCR produces, and individual clusters were sequenced in parallel by one of several technologies [7]. Current NGS platforms include the Roche 454 Genome Sequencer, Illumina's Genome Analyzer, and Applied Biosystems' SOLiD. These platforms can analyze tens to hundreds of millions of DNA fragments simultaneously, generate giga-bases of sequence information from a single run, and have revolutionized SAGE and cDNA sequencing technology [11,12]. For example, the 3' tag Digital Gene Expression (DGE) uses oligo-dT priming for first strand cDNA synthesis, generates libraries that are enriched in the 3' untranslated regions of polyadenylated mRNAs, and produces 20-21 base cDNA tags. It's reported that 99.8% of 21-base tags occur only once in human genome [13], while analyses based on actual sequence information from approximately 16,000 known genes suggest that >75% of 21-base pair tags are expected to occur only once in the human genome, with the remaining tags matching duplicated genes or repeat sequences [13]. The work reported in this manuscript effectively generates 20-base pair tags and the % of tags mapped to multiple locations on genome would be slightly higher. The 3' tag DGE profiling using Illumina Genome Analyzer II generates up to 10-12 million reads from individual libraries of unique, positionally known 20- or 21- base pair 3' cDNA tags. DGE technology generates such extensive sequencing depth-of-coverage that single copy resolution of gene expression quantification should be possible. For example, it's generally accepted that on average there are 350,000 transcripts expressed per cell (Illumina White Paper: mRNA Expression Analysis). Therefore a 10 million tag sequencing experiment by the Illumina GA II would detect 30 tags per transcript expressed at one copy per cell. This technology makes it possible, for the first time, to interrogate low abundance transcripts, which may comprise as much as half of the non-structural RNA within a cell. The alternative to 3' tag DGE sequencing is full-length RNA sequencing (RNA-Seq), which collects both quantitative and qualitative information about the entire transcriptome. RNA-Seq is a powerful tool for identifying expressed polymorphisms as well as differentially expressed splice variants, fusion genes, transcriptional start sites, and polyadenylylation sites [14]. However, the number of reads needed to characterize whole transcript sequences, using RNA-Seq, increases the cost of measuring rare transcripts and may be more economically quantitated with 3' tag DGE profiling. Assessment of the 3' DGE method has been limited. One study used 3' tag DGE approach in mouse hippocampal expression profiling [15], and another paper compared 3' tag DGE with SAGE [15]. More research is needed for evaluation of the sensitivity, dynamic range, reproducibility, and measurement accuracy of 3' tag DGE.

Our work focuses on exploring and validating the applicability of 3' tag DGE to the quantification of transcripts. To this end, we have designed a set of experiments to assess the use of 3' tag DGE as an alternative to microarrays or to RNA-Seq. We used two RNA samples described in the MicroArray Quality Control (MAQC) project [16]: Human Brain Reference RNA (HBRR) and Universal Human Reference RNA (UHRR). The samples, from the exact batches used by the MAQC project, are commercially available to the research community and have been well-characterized by microarray (repeatedly assayed by 7 microarray platforms) and quantitative real time PCR (qPCR, 3 quantitative PCR platforms). Therefore, comparing the profiles of these two RNA samples generated by the 3' tag DGE, by microarray, and by qPCR will provide an excellent frame of reference for evaluation of the sensitivity, dynamic range, reproducibility, and accuracy of 3' tag DGE sequencing.

## Methods

### Library preparation and sequencing

The universal human reference RNA (UHRR) was purchased from Stratagene (catalog no.740000) and the human brain reference RNA (HBRR) was purchased from Ambion/Applied Bioscience (catalog no. AM6051). The 3' tag DGE libraries were constructed from UHRR or HBRR as described in the Illumina DGE protocol. Total RNA (1-2 μg) was fractionated using oligo-dT magnetic beads to yield poly(A+) mRNA. mRNA bound to the beads was then used as a template for first strand cDNA synthesis primed by oligo-dT and the second strand cDNA was consequently synthesized using random primers. Next, the double stranded cDNA covalently attached to oligo-dT beads was digested with *DpnII*. The fragments that remained attached to the beads were ligated to the Illumina GEX DpnII Adapter 1, which includes a *MmeI *recognition site. Therefore, the library preparation protocol allows only one tag per RNA molecule. Digestion with *MmeI *yielded the adapter tag linked to 20 bp of cDNA including 4 bp of the *DpnII *recognition site, which was ligated to GEX Adapter 2 at the site of *MmeI *cleavage. The resulting sequences, tagged on both 5' and 3' ends, were amplified using PCR and purified on 6% Novex TBE PAGE gels. A band corresponding to approximately 85 base pairs was cut from the gel, eluted, and concentrated by precipitation. Integrity of the tagged sequence was confirmed using the Agilent 2100 Bioanalyzer. In addition, an aliquot of each sample was TA cloned into a TOPO vector, a small number of colonies (typically 6-10) were isolated, and plasmid DNA was purified from each. Sanger sequencing was carried out to demonstrate that the libraries were composed of unique clones, each of which contained authentic 3' UTR sequences. Sequencing was carried out at Mayo Advanced Genomics Technology Center DNA sequencing lab using the Illumina Genome Analyzers I and II.

### Image processing and read alignment

Illumina Pipeline Software version 1.0 was used for off-instrument data processing. Images from every sequencing cycle were converted to signal intensities using Illumina Pipeline's FireCrest v.1.9.5. Next, Bustard v.1.9.5 was run to perform base calling using the intensity values and calculate quality scores for every base. The 16-base long reads (excluding the 4-base *DpnII *recognition site) were aligned to *DpnII *tag tables generated by Stowers Institute http://research.stowers-institute.org/microarray/tag_tables/index.html using megaBLAST with word size of 12 and low-complexity region filtering turned off. Only reads that perfectly matched to tag tables without mis-matches and gaps were considered. From this set, reads that could be aligned to the Stowers' repeat tag table were excluded (the repeat tag table contains any reads aligned to ≥ 2 locations, unless all locations are from the same gene). The remaining reads were aligned to the combination of canonical (exonic and splice junction tags from protein-coding transcripts), mitochondrial (tags from any mitochondrion-associated transcripts encoded by both genomic and mitochondrial DNA), and ribosomal (tags from rRNA or tRNA) tag tables. Reads mapping on genes with multiple homologous family members were excluded from our analysis. When there were multiple types of tags aligned to different locations of the same gene, the gene expression levels are represented by the summation of all.

### Microarray data analysis

Five each of Affymetrix Human Genome U133 plus2.0 CEL files for HBRR and UHRR samples from MAQC test site 1 were downloaded from Gene Expression Omnibus database (series accession number GSE5350). The CEL files were pre-processed using GC-RMA[17]. The present, marginal, or absent calls for individual probe sets were calculated by Affymetrix MAS 5.0 algorithm. Only the probe sets with "present" calls in all five samples were used in the comparison with 3' tag DGE data.

### Gene ID mapping between sequencing, Affymetrix microarray, and real-time PCR data

Mapping between Affymetrix probe set ID to ENSEMBL gene was done using the Affymetrix annotation file version na27. In instances where multiple probe sets mapped to a single ENSEMBL identifier, the probe set with the highest expression was selected. The identification of gene IDs for qPCR reagents was extracted from the Affymetrix probe set IDs according to MAQC [18].

### Comparison of UHRR and HBRR 3' tag DGE profiles

Eight UHRR and one HBRR libraries were prepared and compared within sample (HBRR library 1 versus HBRR library 2, etc.) and across samples (UHRR versus HBRR). The gene expression was normalized to tag counts per million total tags (counts per million tags, CPMT) in each lane of a flow cell.

## Results

Our initial analyses focused on defining the reproducibility of 3' tag DGE and determining the depth of sequencing required to achieve a reasonable coverage of the transcriptome. To this end, eight HBRR libraries (L1-L8) and 1 UHRR library (L9) were independently prepared and sequenced in 6 flow cells for a total of 38 flow cell lanes (35 lanes for HBRR and 3 lanes for UHRR) of data using Illumina Genome Analyzer (GA) I and II (Table 1). One lane from each flow cell was used to run the bacteriophage ΦX174 DNA control sample. The lane lay-out for individual sequencing flow-cells (runs) is shown in Table 2.

**Table 1:** Summary of 3' tag digital gene expression libraries including RNA samples, locations of the lab who prepared the libraries, and the library IDs.

RNA	Lab Location	Library ID
HBRR	Florida	L1

HBRR	Florida	L2

HBRR	Florida	L3

HBRR	Minnesota	L4

HBRR	Minnesota	L5

HBRR	Minnesota	L6

HBRR	Minnesota	L7

HBRR	Minnesota	L8

UHRR	Florida	L9

HBRR: Human Brain Reference RNA; UHRR: Universal Human Reference RNA.

**Table 2:** Flow cell lay-out for individual sequencing runs.

Run #	Sequencer	Lane Numbers							
		
		1	2	3	4	5	6	7	8
1	GA I	L1	L1	L2	L2	PhiX	L3	L3	L3

2	GA I	L4	L5	L4	L5	PhiX	L4	L6	L4

3	GA II	L4	L5	L4	PhiX	L5	L4	L6	L4

4	GA II	L4	L4	L5	PhiX	L6	L7	L8	L5

5	GA II	L4	L7	L4	PhiX	L8	L6	L7	L8

6	GA II	L9	---	---	L9	PhiX	---	---	L9

Total of 7 runs were performed on Genome Analyzer I (GA I) and GA II. There are 8 lanes on each flow cell named as Lanes 1-8, one of which was used in each run for a control sample PhiX, the bacteriophage DNA.

### Run Summaries

The summary of run and alignment statistics for all libraries is provided in Additional File 1. One lane of sequencing typically generated 3-5 million reads on the GA I and 6-8 million reads on the GA II. On average 54% of the reads aligned to canonical, 6% aligned to mitochondrial, and <2% aligned to rRNA tag tables. For simplicities of comparisons to MAQC results, we merged the canonical, mitochondrial, and rRNA tag tables into one combined table in this manuscript. The remaining reads aligned to repeat (~7%), non-canonical (~3%), non-coding (~0.02%), and intergenic (~14%) tag tables. Approximately 15% of reads did not align to the reference tables.

### Reproducibility

**Figure 1:**
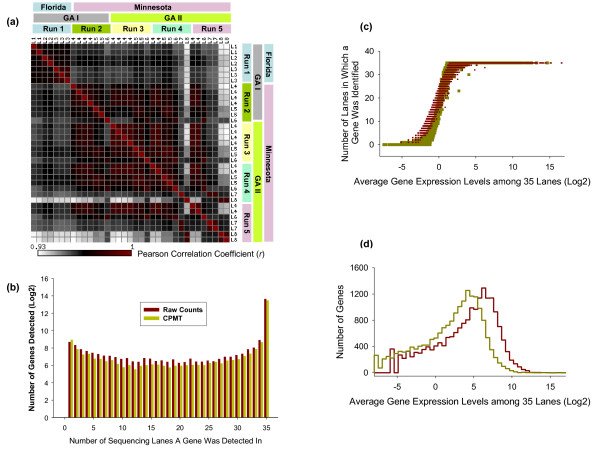
**Reproducibility of 3' tag digital gene expression profiling**. 1a - Pearson correlation coefficient matrix of all 8 HBRR libraries prepared at two Mayo locations (Minnesota and Florida), sequenced in 35 lanes of 5 different runs on two generations of Genome Analyzer (I and II). The rows and columns are all 35 lanes in 5 HBRR sequencing runs, and named using the corresponding library names (L1-L8). The same libraries in each run have been grouped together for visual benefits. The correlation coefficient was calculated using Log2 transformed tag counts. The tag counts of zero were coded as missing data. The actual numbers of the correlation coefficient are listed in the Additional File 2. The color in each squire of the matrix reflects the pair-wise lane-to-lane degree of correlation of gene expression levels; 1b - Concordance of gene detection. Gene expression levels are represented by the raw number of reads (dark red) and number of reads per million (yellow). More than 70% of the genes were repeatedly detected in all 35 lanes. 1c - The relationship between gene detection and expression levels. Genes that were detected in less than 35 lanes were lower expressed at levels of 1-2 CPMT, or 0.35-0.7 copies per cell; 1d - The histogram of gene expression levels.

As shown in Table 1, eight HBRR libraries were sequenced in order to assess reproducibility between the Genome Analyzer (GA) II and the older GA I, reproducibility within- and between- sequencing runs, and reproducibility between library preparations performed at two Mayo locations (Minnesota and Florida). First, we looked at the correlations of the gene expression levels. Figure 1a shows the pair-wise lane-to-lane Pearson Correlation Coefficient of the gene expression levels. Except for library 8 (L8) and to a lesser extent Library 6 (L6), we observed very good correlations (r > 0.95) of HBRR expression between technical replicates (same library, same run, different lanes) and biological replicates (different libraries, same or different runs). The correlations of libraries run on GA I and GA II were also > 0.95. A complete table containing the raw gene tag counts (Log2 scale) and full Pearson correlation coefficient matrix, for all sequencing runs, can be found in Additional File 2.

Second, we looked at the concordance/reproducibility of gene identification across different lanes and different libraries. We asked the question: "if a gene was identified in one lane or one library, how often was it reproducibly identified in a different lane of the same library, or in a different library of the same RNA sample"? We defined a gene as being "identified" when it could be associated to at least one tag, or 1 CPMT. A total of 18,000 genes with at least one tag are identified from the combined analysis of the reads included in the 35 lanes of HBRR sequencing. Of these genes, 12,825 (71.25%) are repeatedly identified in all lanes and all libraries, with the other 5,175 (28.75%) detected by less number of lanes and/or libraries (Figure 1b). The small peak in the histogram around genes identified by 1-5 lanes reflects the groups of extremely low expressed genes whose expression levels are around the detection sensitivity threshold of DGE at current sequencing depth. As shown in Figure 1c and 1d, which illustrate the relationship between gene expression level and number of lanes in which the gene was identified (Figure 1c), and the histogram of gene expressions (Figure 1d), all genes not consistently identified in all 35 lanes had expression levels between 1-2 CPMT, or 0.35-0.7 copies per cell. Since late 2008 and early 2009 when these libraries were sequenced, the throughput of one lane of sequencing on GA II has increased to 15-20 million reads, due to new sequencing chemistry and new version of the Illumina Pipeline Software (v1.4). Therefore, we expect that the percentage of the genes repeatedly detected across lanes to be greatly improved. We calculated that theoretically up to 90% of the current 18,000 genes would be detected across all 35 lanes with this much higher sequencing capacity.

### Tag to gene relationship

**Figure 2:**
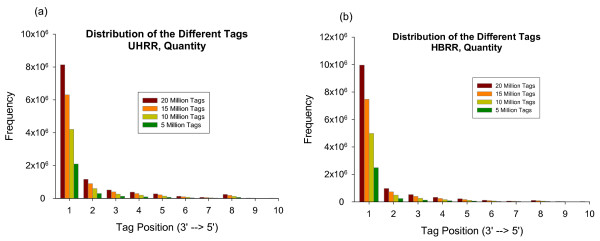
**Distribution of tag frequency per DpnII digestion site (ordered 3' to 5') for samples UHRR (2a) and HBRR (2b)**.

The enzymatic digestion protocol used in the 3' tag DGE approach is designed to capture the digestion site most proximal to a transcript polyadenylylation site. If one assumes complete *DpnII *digestion during library preparation and assumes a single polyadenylylation site per gene, there should be theoretically only one type of tag per transcript. However, according to the run summary data in Additional File 1, there were on average more than 3 unique tags (reads) per unique gene identified in the HBRR and UHRR samples. In fact, when we mapped tags to all possible enzymatic digestion sites, there were up to 50 unique tags per gene. For example, the analysis of 3 libraries (L1-L3) of HBRR indicates that the number of unique tags per gene range from 1-46 tags with a mean of 4 and a median of 3 (data not shown). Figure 2 shows frequency distribution plots of tags per digestion site (referenced relative to the transcript 3' end), as a function of the total number of reads analyzed. From the pool of more than 200 million reads collected from the 35 HBRR flow cell lanes, we randomly selected 5, 10, 15, or 20 million reads for the incremental analysis. On average, in both HBRR and UHRR samples, the 3' most *DpnII *digestion site (position 1) accounts for 70-80% of the total mapped tags, with an exponential decrease in the number of tags observed as the digestion site becomes closer to the 5' end region. However, there were multiple instances where the tag count distribution for an individual transcript did not fit this profile. This is the case for gene PGK1 (Additional File 3, figure S3) where the two most abundant tags were the 1^st ^and 4^th ^tags from the 3' end, each corresponding to a known poly-adenylation site. This un-common profile is therefore more likely due to the presence of multiple poly-adenylation sites, although we couldn't exclude that there might be concurrent incomplete digestion by *DpnII*. We also briefly discussed other genes whose most abundant tags were not the 3' most tags in Additional File 3, figure S4.

### Transcriptome coverage vs. sequencing depth

**Figure 3:**
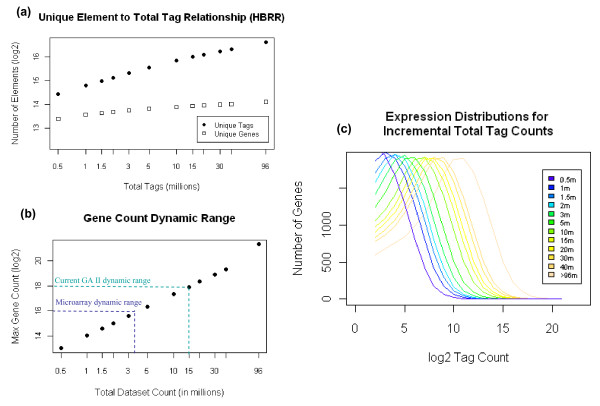
**Relationship in HBRR between increasing number of raw sequences analyzed (0.5 to 96 million) and the number of unique 3a -- tags and genes identified, 3b -- the increase in dynamic range of gene expression measurement, and 3c -- the impact on the distribution of gene expression**.

In order to estimate the sequencing depth needed for sufficient transcriptome coverage, we studied the cumulative benefit of sequencing an increasing number of reads. Different sequencing depths were simulated by randomly selecting 0.5, 1, 1.5, 2, 3, 5, 10, 15, 20, 30, 40, or all 96 million tags from the HBRR libraries. The number of unique tags, the number of unique genes, and the observed dynamic range were calculated at each sequencing depth (Figure 3). All three parameters increase as the total number of tags sequenced increases. However, the observation of novel tag sequences increased at substantially faster rate then the observation of novel genes (Figure 3a), as less abundant gene tag types started to be detected with the increase in sequencing depth.

Figure 3b plots the tag counts of the highest expressed gene obtained from the 3' tag DGE at different sequencing depths, which increase linearly as more sequences are included in the analysis. Since the total number of genes sequenced increases with higher sequencing depth, plotting the tag counts of the highest expressed gene at any given sequencing depth effectively plots the dynamic range of the experiment. Although detection of very abundant transcripts is not a challenge for any of the available technologies, accurate quantification of such mRNAs is another matter. All hybridization-based technologies are susceptible to saturation, which limits the ability to compare the level of expression of transcripts that are expressed at very high levels in certain samples. Sequence based quantification methods are theoretically not saturable and should therefore exhibit a higher limit to the dynamic range. According to literature, the highest hybridization signals of an Affymetrix GeneChip array is around 50,000 intensity units (15-16 log _2_), without scaling. The actual upper limit of the dynamic range of microarrays is probably somewhat narrower if one considers optical background from the scanner and non-specific hybridizations, as well as the signal saturation. However, if one accepts the assertion that the upper limit of the dynamic range of microarrays is between 15 and 16 at Log2 scale, then the limit is comparable to that of one lane of 3' tag DGE using the older version GA I platform, which generates 3-5 million tags per lane (Figure 3b). The newer GA II platform generates 6-8 million tags per lane (as seen with our data from the GA II instrument), and the most recent iteration of the GA IIx is predicted to generate ~15-20 million tags per lane (using Illumina Pipeline Software version 1.4, Figure 3b). Increasing the total number of sequences analyzed increases the upper limit of the dynamic range of the DGE method, and Figure 3c clearly illustrates that the distribution of the higher expressed genes is not skewed, nor do the signals appear to reach a saturation point as additional sequences are accumulated. We conclude, therefore, that DGE technology has greater power for quantification of high abundance transcripts, compared to microarray technology.

### 3' DGE to quantitative real-time PCR (qPCR) comparisons

**Figure 4:**
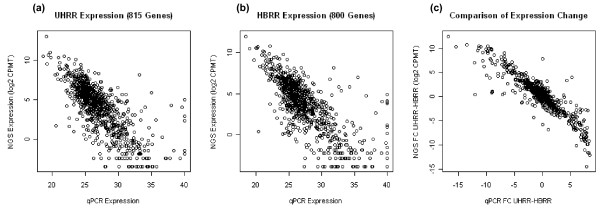
**Scatter plot of gene expression level measurements using the 3' tag DGE and qPCR technologies, for (4a) UHRR sample, (4b) HBRR sample, and (4c) UHRR *vs*. HBRR differential expression**. The gene expression levels from qPCR are represented by PCR cycle number, and the expression levels from DGE are represented by Log2 of CPMT (counts per million tags)

To assess the accuracy of 3' tag DGE, gene expression levels were benchmarked against MAQC sample TaqMan qPCR data [19]. There were 815 Ensembl genes in the UHRR data set and 800 Ensembl genes in the HBRR data set where expression results were available from both the 3' tag DGE and qPCR analyses. Pearson correlations of -0.740 (p-value < 2.2 e-16) and -0.746 (p-value < 2.2 e-16) and Spearman correlations of -0.765 (p-value < 2.2 e-16) and -0.747 (p-value < 2.2 e-16) were obtained between DGE and qPCR for UHRR and HBRR expression results, respectively (Figure 4a and 4b). The negative correlation reflects the fact that qPCR data is measured in amplification cycles, with larger values associated to lower expression. The correlation in absolute expression levels was skewed by a small number of outliers most of which were low-expressed genes (lower right corner of Figures 4a and 4b). The discrepancy in expression quantitation for these outliers genes may reflect limitations in the measurement range for both technologies. Since measurement scales differ between technologies, the differential expression between UHRR and HBRR samples was also compared. The Pearson and Spearman correlations between fold change values for DGE and qPCR are -0.902 (p-value < 2.2e-16) and -0.876 (p-value < 2.2e-16) respectively, as is illustrated in Figure 4c. According to the MAQC publication [17] the correlation of fold changes between Affymetrix microarray and qPCR is 0.92 which is slightly higher. The high correlation of differential expression measured by NGS and gold-standard qPCR validates and demonstrates value in using NGS DGE for gene expression quantitation.

### NGS vs. Affymetrix U133Plus 2.0 microarray in gene detection

**Figure 5:**
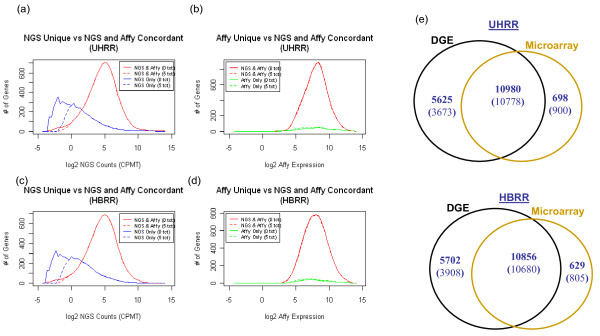
**ENSEMBL gene expression distribution separated by method of detection and sample analyzed: expression distribution in DGE counts for genes identified in (a) UHRR and (c) HBRR, by both DGE and Affymetrix microarray (red line), as well as by DGE alone (blue line); expression distribution in Affymetrix microarray expression values for genes identified in (b) UHRR and (d) HBRR, by both DGE and Affymetrix microarray (red line), as well as by Affymetrix alone (green line)**. The differential expression between UHRR and HBRR for genes identified by: (e) both DGE and Affymetrix, as well as, NGS alone, in NGS counts; (f) both NGS and Affymetrix, as well as, Affymetrix only, in Affymetrix microarray expression values. (g) Comparison of the number of expressed genes detected by DGE and microarrays. Values for relaxed (at least one read) and stringent (at least five reads) DGE parameters are in bold or in brackets, respectively.

A comparison between DGE and Affymetrix U133 Plus 2.0 microarray was performed since microarray technology has been the primary method for measuring genome-wide gene expression levels. Additional File 4 summarizes the observed ENSEMBL gene expression in the Affymetrix and NGS data. There were 17,303 UHRR and 17,187 HBRR unique ENSEMBL genes identified by the two technologies combined. Sixty-three percent of these genes were commonly observed, 33% were only observed by 3' tag DGE without a minimum count threshold, and 4% were only observed by Affymetrix microarray. When applying a >5-count threshold in the UHRR data set, the total number of observed genes decreased by ~13% (Figure 5e); the percent of total genes detected by DGE and microarray, by DGE alone, and by microarray alone is respectively 70%, 24%, and 6%. Smoothed histogram plots shown in Figure 5a-d provide additional information regarding the expression characteristics of the genes uniquely identified by each technology. UHRR or HBRR genes identified by both technologies have normal distributions centered on log_2 _5.3 CPMT DGE counts or log_2 _8.3 Affymetrix expression level. Genes identified by DGE (>0 or >5 counts) but not by Affymetrix microarray are predominantly low in abundance, with the total distribution of expression represented by a decaying function peaking at genes with a single count and running out to a few genes with log_2_16 counts. Conversely, the genes identified by Affymetrix microarray but not by NGS were more uniformly distributed and did not exhibit preference towards a specific expression level. The ability for DGE to quantify higher numbers of low-expressed genes than the Affymetrix microarray was evident in both HBRR an UHRR samples.

The reproducibility of gene expression levels between DGE and Affymetrix microarray was also evaluated. Additional File 3 figure S5 contains scatter plots of DGE expression versus Affymetrix expression for 10,980 ENSEMBL genes in the UHRR sample, 10,856 in the HBRR sample, and the UHRR-HBRR differential expression of 9512 genes. The Pearson correlations for the UHRR, HBRR, and UHRR-HBRR differential expression, were respectively 0.668, 0.657, and 0.895. These correlations reflect the observed agreement in expression levels determined by the two technologies. However, a subset of genes with low-expression determined by DGE has a wide range of expression levels measured by Affymetrix microarray. These genes may be reflective of cross-hybridization or incorrect annotation in the microarray data, or genes that were poorly sequenced and incorrectly measured using DGE.

## Discussion and conclusion

Massively parallel sequencing for transcriptome profiling generates digital counts of gene expression levels compared to the "analog" hybridization signals from the traditional microarray or quantitative PCR methods. Our analysis highlights the precise nature of DGE profiling, as demonstrated by the high reproducibility between technical replicates of the same library run in different lanes, and between biological replicates of different libraries on the same or different flow cells runs. The correlations between technical replicates were >0.97. Although variability between biological replicates of two different libraries (L6 and L8) was less than 0.95, it is likely that the variance came from library construction and not the sequencing process, since the correlations between biological replicates of the libraries constructed at one lab (Florida) were excellent (> 0.95, Additional File 2). We also demonstrated that DGE profiling is accurate. The measurement of fold changes of genes between HBRR and UHRR was highly correlated with data obtained from qPCR. This correlation is similar to that between microarrays and qPCR.

With 20 million tags each from HBRR and UHRR library, which is the current sequencing throughput of one lane of sequencing on GA II, DGE detected 10-20% more transcripts than microarrays, a majority of which were expressed at levels below the sensitivity threshold of microarray platforms. The detection of the lower-expressed genes and the wider dynamic range have been shown to be the main advantages by DGE compared to microarray analysis, since other parameters evaluated between the two platform were mostly comparable. It has been suggested that lower expressed transcripts may account for nearly half of all transcripts in a cell, and play critical but currently undefined roles in pathology and physiology. DGE's ability to quantify these transcripts may open new horizons for the application of genomic profiling to translational research. In addition, DGE may lead to the discovery of new functional genomic regions. For example, 13-15% of the reads from our DGE libraries aligned to intergenic regions which may be related to novel transcripts. However, since no DNase treatment was performed during RNA extraction, these un-mapped reads could also be from the contamination of the genomic DNA although this scenario is less likely because of the selection of PolyA+ RNAs during library construction.

One limitation of 3' DGE is that using a particular enzyme such as *DpnII *for library preparation requires the presence of *DpnII *restriction site(s) in the mRNA. Some transcripts, even though highly expressed, may lack the *DpnII *site(s) and therefore not represented in the libraries. Among all 43,569 transcripts in Human RefSeq RNA database (version of Aug 24, 2009), 2,912 (6.68%) don't have a *DpnII *site. Among 28,061 mature RNAs with accession numbers starting with NM_ (excluding XR_, NR_ and XM_ accession numbers), 571 (2.04%) don't have a *DpnII *site. Additional File 5 lists all Human RefSeq RNAs and the number of *DpnII *sites within each molecule. In addition, the non-polyadenylated transcripts are not represented in the current libraries. In an effort to measure both mRNA and non-polyadenylated RNAs (data not shown), we tried to treat total RNAs with RiboMinus™ Transcriptome Isolation Kit (Invitrogen) prior to library preparation to deplete 18S and 28S rRNAs and to enrich polyadenylated mRNA, non-polyadenylated RNA, pre-processed RNA, tRNA, and small rRNAs (5S rRNA, 5.8S rRNA). However, the sequencing of RiboMinus libraries revealed that ~70% of the reads were rRNAs (data not shown). Therefore, we made a decision to prepare polyadenylated mRNA libraries for the current study to avoid wasting sequencing depth on rRNAs.

On the other hand, some of the genes that were detected by Affymetrix only may only have *DpnII *tags that were mapped to multiple genes. These tags were excluded from our analysis. We point the readers to the web site of the Stowers Institute where these redundantly mapped tags were recorded http://research.stowers-institute.org/microarray/tag_tables/downloads.html.

Several challenges currently limit the adoption of 3' DGE in replacement of microarrays. The higher cost per sample and low sample throughput per run limit the sample size in a study. Currently 3' tag DGE analysis costs ~$1200 per lane of sequencing including library construction. It also has long individual run times, sequencing only ~12 bases per day, and sample preparation is significantly more difficult and time consuming than that of microarray. Finally, the bioinformatics challenges associated with 3'DGE analysis are significant, including storage, archiving, and retrieval of the vast volume of data, and development of algorithms to assemble and align sequence reads as short as 35-40 nucleotides [19]. However, advances in these new sequencing technologies will substantially increase sample throughput, leveraging techniques such as multiplexing that could reduce the cost of sequencing per sample. Bioinformatics challenges are currently being addressed, faster and more powerful alignment tools being developed.

We are aware that RNA-Seq enables a more extensive profiling of the transcriptome by facilitating the discovery of fusion genes [20], detection and quantification of alternative splice forms, and characterization of expressed mutations and polymorphisms [14]. However, in order to comprehensively sequence the full length of all transcripts, the sequencing depth of RNA-seq needs to be significantly greater compared to that of 3' tag DGE profiling. It was estimated that at least 40 million reads (compared to < 5 million reads required for DGE) needs to be sequenced from a single library to achieve 90% coverage of the transcriptome [10] making RNA-seq even more financially demanding that 3' tag DGE. In addition, the increased complexity of the data poses even greater analytic challenges. Therefore, the advantages of 3' tag DGE over microarray in profiling lower-expressed genes and measuring with greater dynamic range make it arguably attractive for use in today's medical and biological research.

## Authors' contributions

EAT, DIS, and EDW performed sample preparation and sequencing. YWA, EWK, SM, ALO, and TMT carried out the data analysis. EAT, EAP, GAP and JPAK coordinated the execution of the project. YWA, EWK and EAT wrote the manuscript. All authors have read and approved the final manuscript.

## Supplementary Material

Additional file 1 **Table S1, which is the summary of runs and alignment statistics for all libraries**. Click here for file

Additional file 2 **Table S2, a complete table containing the raw gene tag counts (Log 2 scale) and full Pearson correlation coefficient matrix for all lanes and flow cells of all sequencing runs**. Click here for file

Additional file 3 **Figures S3 - S5 as described in the manuscript**. Click here for file

Additional file 4 **Table S6, representation of expressed ENSEMBL genes in the NGS DGE and Affymetrix U133Plus 2.0 expression data sets**. Click here for file

Additional file 5 **Table S7, the number of *DpnII *sites in each of the Human RefSeq RNAs**. Click here for file
