# Supplementary material for: 3' tag digital gene expression profiling of human brain and universal reference RNA using Illumina Genome Analyzer
Source: BMC Genomics. 2009 Nov 16;10:531. doi: 10.1186/1471-2164-10-531 (PMC2781828; doi:10.1186/1471-2164-10-531)
Supplement: Additional file 3 — Figures S3 - S5 as described in the manuscript. [file 1471-2164-10-531-S3.PPT]

## Slide 1
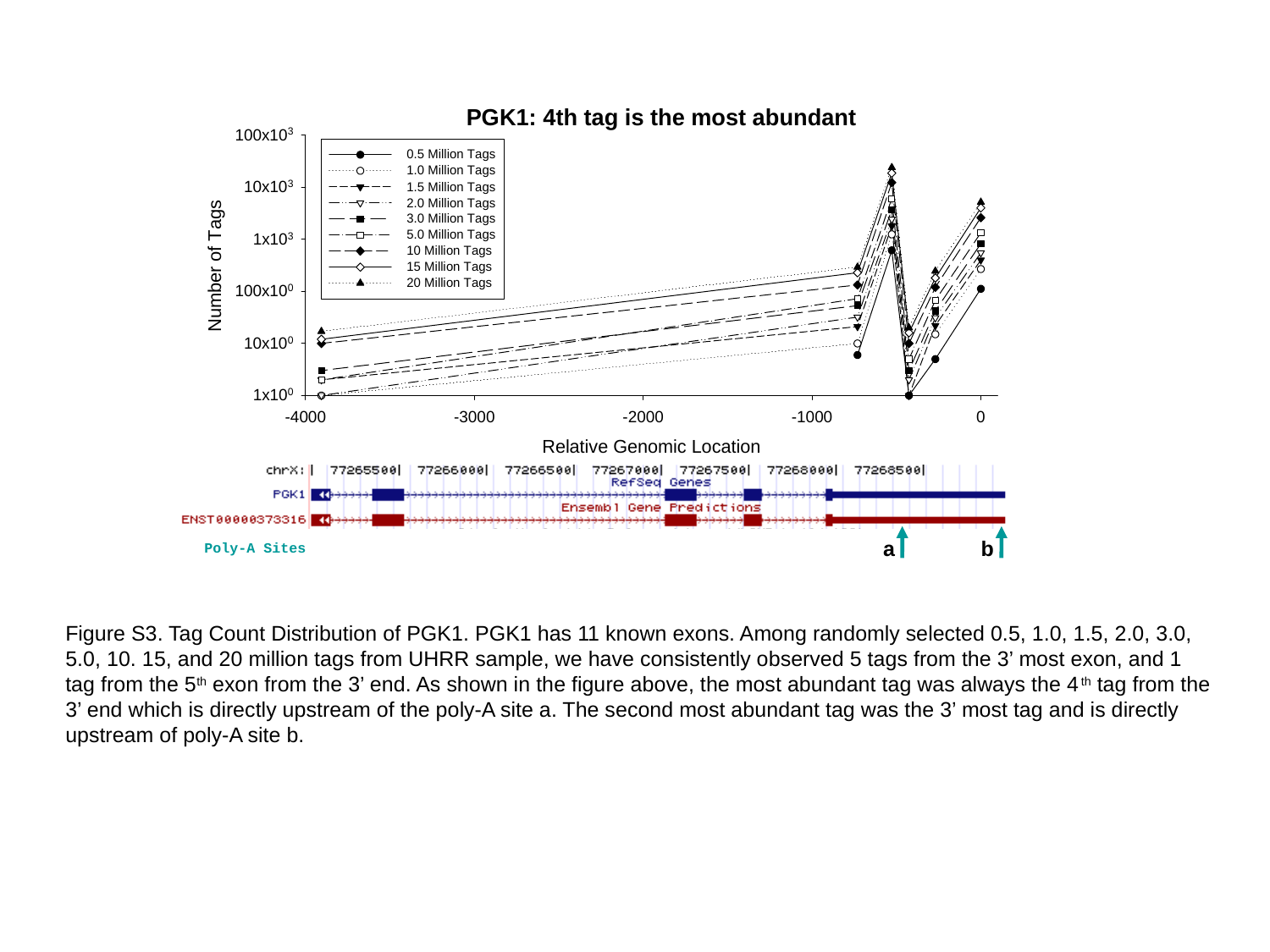

a
b
Poly-A Sites
Figure S3. Tag Count Distribution of PGK1. PGK1 has 11 known exons. Among randomly selected 0.5, 1.0, 1.5, 2.0, 3.0, 5.0, 10. 15, and 20 million tags from UHRR sample, we have consistently observed 5 tags from the 3’ most exon, and 1 tag from the 5th exon from the 3’ end. As shown in the figure above, the most abundant tag was always the 4th tag from the 3’ end which is directly upstream of the poly-A site a. The second most abundant tag was the 3’ most tag and is directly upstream of poly-A site b.

## Slide 2
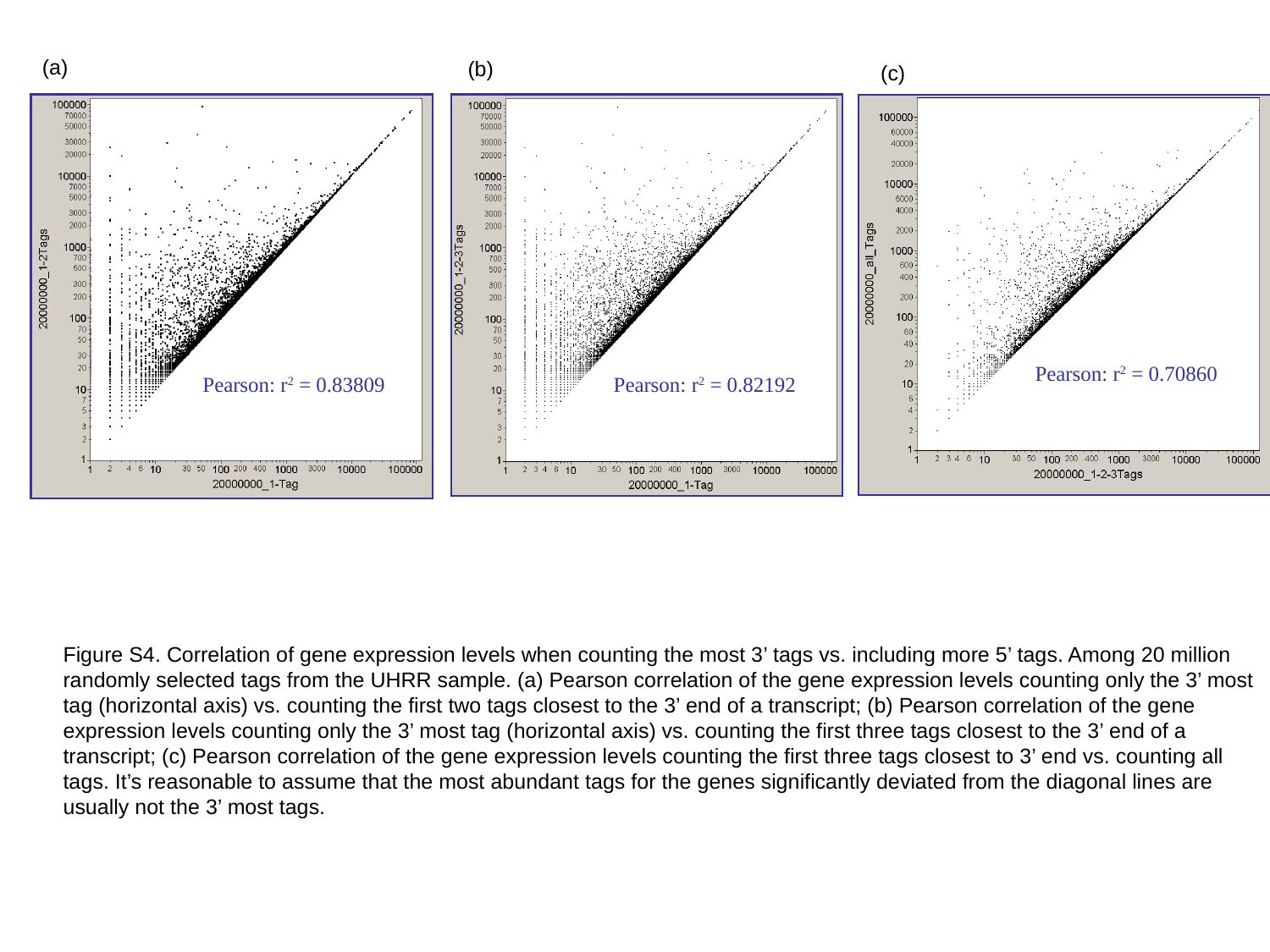

(a)
(b)
(c)
Pearson: r2 = 0.70860
Pearson: r2 = 0.83809
Pearson: r2 = 0.82192
Figure S4. Correlation of gene expression levels when counting the most 3’ tags vs. including more 5’ tags. Among 20 million randomly selected tags from the UHRR sample. (a) Pearson correlation of the gene expression levels counting only the 3’ most tag (horizontal axis) vs. counting the first two tags closest to the 3’ end of a transcript; (b) Pearson correlation of the gene expression levels counting only the 3’ most tag (horizontal axis) vs. counting the first three tags closest to the 3’ end of a transcript; (c) Pearson correlation of the gene expression levels counting the first three tags closest to 3’ end vs. counting all tags. It’s reasonable to assume that the most abundant tags for the genes significantly deviated from the diagonal lines are usually not the 3’ most tags.

## Slide 3
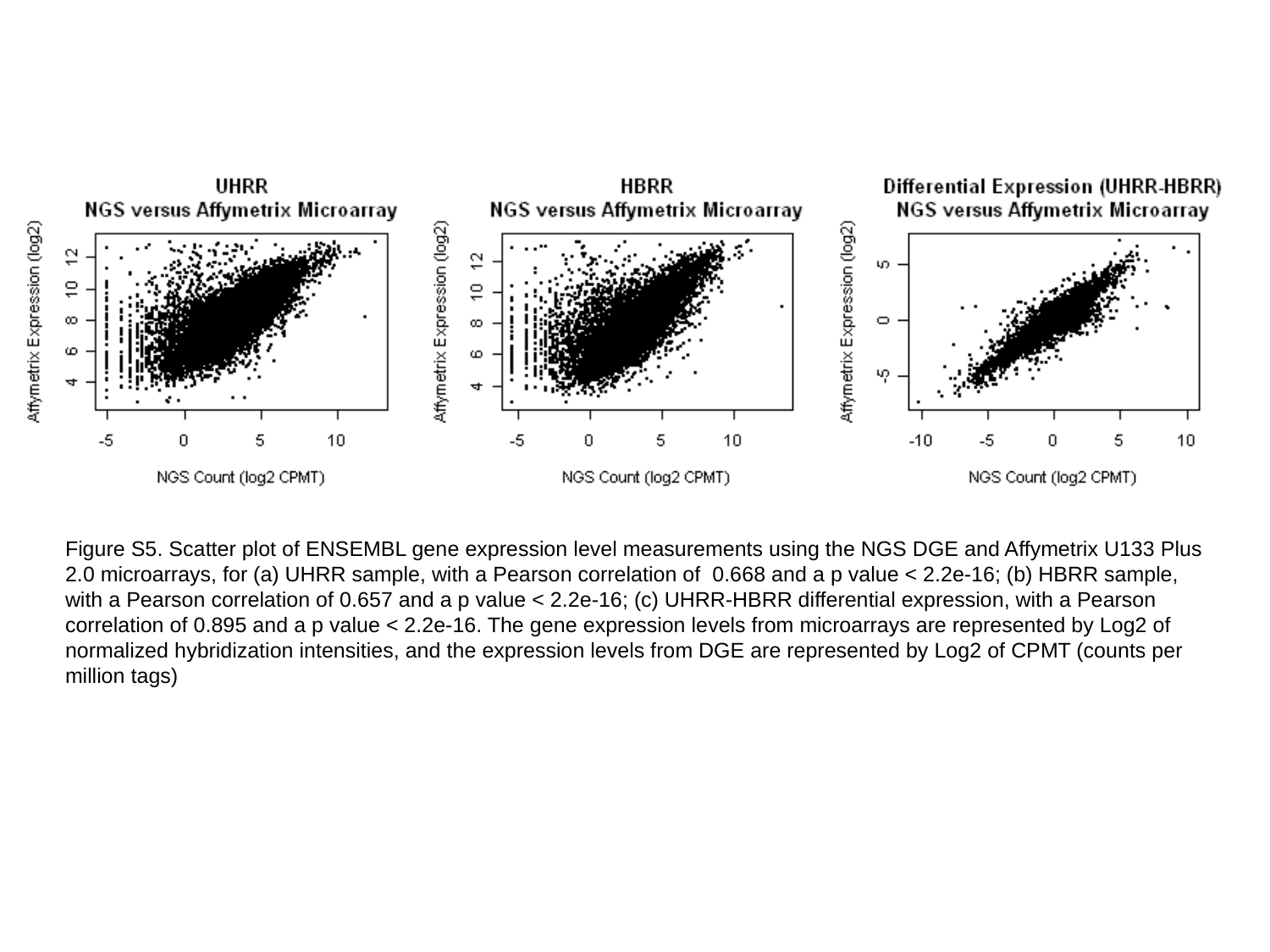

Figure S5. Scatter plot of ENSEMBL gene expression level measurements using the NGS DGE and Affymetrix U133 Plus 2.0 microarrays, for (a) UHRR sample, with a Pearson correlation of 0.668 and a p value < 2.2e-16; (b) HBRR sample, with a Pearson correlation of 0.657 and a p value < 2.2e-16; (c) UHRR-HBRR differential expression, with a Pearson correlation of 0.895 and a p value < 2.2e-16. The gene expression levels from microarrays are represented by Log2 of normalized hybridization intensities, and the expression levels from DGE are represented by Log2 of CPMT (counts per million tags)
