# Supplementary material for: 3' tag digital gene expression profiling of human brain and universal reference RNA using Illumina Genome Analyzer
Source: BMC Genomics. 2009 Nov 16;10:531. doi: 10.1186/1471-2164-10-531 (PMC2781828; doi:10.1186/1471-2164-10-531)
Supplement: Additional file 4 — Table S6, representation of expressed ENSEMBL genes in the NGS DGE and Affymetrix U133Plus 2.0 expression data sets. [file 1471-2164-10-531-S4.DOC]

Table S6

**Table S6** Representation of expressed ENSEMBL genes in the NGS DGE and Affymetrix U133Plus 2.0 expression datasets. NGS count values were computed by summing counts from 3 lanes of sequencing. Analysis was performed with multiple minimum count thresholds for determining NGS gene expression (0, 16,10,5)

|  | Expressed ENSEMBL Genes | | |
| --- | --- | --- | --- |
|  | NGS and Affymetrix | NGS Only | Affymetrix Only |
| UHRR | 10,980 (10467)(10633)(10778) | 5625 (2467)(2957)(3673) | 698 (1211)(1045)(900) |
| HBRR | 10,856 (10392)(10554)(10680) | 5,702 (2831)(3283)(3908) | 629 (1093)(931)(805) |
